# Mechanical and Acoustic Emission Characteristics of Coal-like Rock Specimens under Static Direct Shear and Dynamic Normal Load

**DOI:** 10.3390/ma15196546

**Published:** 2022-09-21

**Authors:** Jun Guo, Luyang Yu, Zhijie Wen, Guorui Feng, Jinwen Bai, Xiaoze Wen, Tingye Qi, Ruipeng Qian, Linjun Zhu, Xingchen Guo, Xincheng Mi

**Affiliations:** 1 College of Mining Technology, Taiyuan University of Technology, Taiyuan 030024, China; 2 Key Laboratory of Shanxi Province for Mine Rock Strata Control and Disaster Prevention, Taiyuan 030024, China; 3 Shanxi Province Coal-Based Resources Green and High-Efficiency Development Engineering Center, Taiyuan 030024, China; 4 Shanxi Coking Coal Group Co., Ltd., Taiyuan 030024, China; 5 State Key Laboratory of Strata Intelligent Control and Green Mining Co-Founded by Shandong Province and the Ministry of Science and Technology, Shandong University of Science and Technology, 579 Qianwangang Road, Economic and Technological Development Zone, Qingdao 266590, China

**Keywords:** dynamic and constant load coupling action, shear failure, mechanical properties, acoustic emission, coal-like rock materials

## Abstract

In underground engineering, shear failure is a common failure type in coal-rock mass under medium and low strain-rate disturbance loads. Analyzing the shear failure mechanical properties of coal-rock mass under dynamic normal load is significant. In order to reveal the influence of disturbance load on the shear mechanical properties of coal rock, a dynamic and static load coupling electro-hydraulic servo testing machine was used to conduct the shear tests of coal-like rock materials under dynamic and constant normal load. The amplitude of dynamic load is 10 kN and the frequency is 5 Hz. The damage process of the specimens was detected by the acoustic emission (AE) detection system. The results imply that the shear failure process of coal-like rock materials under constant normal load can be divided into four stages. The normal disturbance decreased the shear strength of the specimens and increased the shear modulus of the specimens. With the increase in normal load, the influence of disturbance on the shear strength of the specimen decreased. By analyzing the AE parameters, it was found that the dynamic load made the internal damage of the specimen more severe during the shear failure process. The damage variable was calculated by AE cumulative energy, and the damage evolution was divided into three stages. The shear failure mechanism of the specimen was judged by RA (rise time/amplitude) and AF (average frequency). It was found that from the elastic deformation stage to the unstable development fracture stage, the proportion of shear fracture increased. When the dynamic normal load was 10 kN and 30 kN, the fracture was mainly shear fracture; When the dynamic normal load was 50 kN, the fracture was mainly tensile or mixed fracture. The dynamic normal load affects the shear strength and failure mechanism. Therefore, the influence of disturbance load on coal-rock mass strength cannot be ignored in underground engineering.

## 1. Introduction

In deep underground engineering, the geological environment is complex and engineering disasters are serious. Mining methods and changes in the stress states of rocks can induce seismic events [1]. The formation and distribution of zones of higher stress concentrations require special attention in order to prevent a dynamic phenomenon [2]. With the increase in mining depth and volume, the frequency and scale of mining induced seismic events will increase [3]. Coal-rock mass is usually affected by dynamic loads. Especially in the process of coal mining, in addition to the influence of high ground-stress, deep underground engineering is also affected by the disturbance caused by frequent blasting and mechanized drilling during construction. In fact, coal-rock mass is in the combined stress state of “static pre-loading + dynamic disturbance”. Rock mass instability often occurs under the coupling of dynamic and static loads. To achieve safe and efficient mining, it is necessary to fully understand coal-rock mechanical properties under complex stress conditions. In order to understand the mechanical properties of rock, Cai [4] studied the visualization of rock mass classification to provide a basis for estimating deformation and strength characteristics. Tibbett [5] combined large datasets with virtual reality science visualization (VRSV) to understand complex caves. The study of deep coal-rock mechanics has become a topic of general interest. Many researchers have begun to focus on the behavior of coal rock under dynamic load, fatigue load (cyclic load) and different strain rates. The study of coal-rock behavior under dynamic conditions is of special significance because different dynamic conditions significantly affect mechanics properties [6,7,8,9]. Liang [10] divided the loading strain-rate of rock test into the static loading test, the quasi dynamic loading test and the dynamic loading test. The seismic waveform load test is a medium strain-rate loading test that shows the mechanical characteristics of rock under dynamic load. The dynamic action of the surrounding rock of the mining field is mainly manifested in the shock wave action close to the vibration source, the stress wave action at a medium distance from the vibration source and the seismic wave action far away from the vibration source [11].

To study these questions, different strain-rate tests need the use of different equipment. For instance, servo-hydraulic machines are for quasi-static loading, the split Hopkinson pressure bar (SHPB) is for high strain rate and plate impact techniques have been employed for very high strain rate (VHSR), etc. [12] Many researchers have conducted in-depth research on the problem of rock failure under combined dynamic and static loading or dynamic disturbance. Gao [13] studied the influence of engineering disturbance on the deep in-situ rock mechanical properties. With the increase in depth, the failure mode of coal and rock changes. Gong [14,15] studied the dynamic failure characteristics of sandstone subjected to coupled static and dynamic loads with a modified 3D SHPB apparatus. Cyclic loading and unloading with different high-disturbance frequency tests were performed with the MTS Landmark electro-hydraulic servo testing machine to discover the effects of toughness increasing and decreasing on hard rock fracturing [16]. Li [17] established a coupled static–dynamic constitutive model with intermediate strain rate by combining a static damage model and viscoelastic model. Liu [18] studied the effects of cyclic dynamic loading on the mechanical properties of intact rock samples under confining pressure conditions. Bagde [19] found that the loading frequency and amplitude influenced the dynamic fatigue strength and the dynamic modulus of rock in dynamic cyclic loading conditions.

Shear failure is one of the basic coal-rock mass failure modes. Many scholars have conducted extensive research on the shear failure mechanism through the laboratory direct shear test (DST) of rock specimens [20,21,22,23]. Cheng [24] conducted direct shear tests under different normal stresses with a RDS-200 rock direct shear test system, and established the shear deformation constitutive model of sandstone with irregular joints by using piecewise function. Huang [25] studied the shear behavior of rock under tensile stresses and compression stress, and analyzed the characteristics of rock deformation, strength and fracture surface. The peak shear strength of sandstone shows an approximately linear variation in the compression region. The existing rock material direct shear test studies were conducted mainly under constant normal loads, while relevant studies using dynamic normal loads are fewer. Dang [26,27] studied the mechanical properties of planar joints under dynamic normal load (DNL) with different frequencies and amplitudes with the GS-1000 shear box device. However, the existing rock mechanics or rock dynamics can not be directly used to explain the shear mechanical properties under disturbance. Therefore, it is of great significance to understand the shear mechanical properties and failure mechanism of coal rock under dynamic and static loads for safety mining and for effectively utilizing underground space.

Because coal is a heterogeneous brittle material, when it is sampled in-situ, the coal mass can be damaged. Transportation and sample preparation can damage the coal mass again and affect the test results. Therefore, many scholars use coal-like materials to replace coal-rock mass for tests. Li [28] used sand, cement and corn starch to make materials similar to coal rock for “solid-gas coupling” simulation experiments. Li [29] and Zhai [30] used sand, cement and plaster to make coal-like rock materials to replace real coal for tests. The selection principle for materials similar to coal-rock mass was proposed.

Acoustic emission monitoring is a testing technology. In the process of coal-rock failure, the initiation, propagation and coalescence of internal cracks can produce AE signals. Its signal characteristics are closely related to the coal-rock fracture mechanism, which can better describe its damage and evolution characteristics [31]. By detecting and analyzing these acoustic emission signals, the fracture mechanism and dynamic response characteristics of rocks can be analyzed [32]. Liu conducted graded cyclic loading and unloading tests to reveal the AE characteristics of metasandstone under graded cyclic loading and unloading [33]. Zhao conducted triaxial compression with a permeability test of limestone and used AE technology to monitor the initiation and propagation of microcracks during deformation and failure [34]. Cheon [35], based on the characteristics of acoustic emission parameters, proposed the criteria for evaluating rock fracture type and damage degree, and the method is used to monitor and evaluate the shear failure and damage degree of rock slope. Meng [36] performed direct shear tests under constant normal load conditions on artificial splitting cement mortar fractures with the RMT150C experimental system, and investigated variations in AE parameters with shear stress. AE parameters can characterize the current fracture states of materials so that they can be used as precursor information pertaining to material failure [37]. Zha used AE parameters to study the rock failure process under different loading modes, and defined different precursor points [38]. Wu considered that a sudden increase in the AE value can be a precursor to rock destruction [39]. Aggelis [40] and Wang [41] proposed some AE indices to classified fracture mechanisms. Smaller RA value and lager AF value correspond to the tensile mode, and larger RA value and smaller AF value correspond to the shear mode. Ohno and Ohtsu [42] studied the failure mechanism of concrete. The shear and tensile failures are divided by the split line AF/RA = 80. The results are similar to those of SiGMA moment tensor analysis. Zhou [43] performed lateral disturbance failure tests of granite specimens under different static stresses. By analyzing the RA values, it was found that the disturbance failure modes of rocks under different static stresses were different.

Therefore, in order to study the shear mechanical properties of coal under medium and low strain-rate disturbance loads, the shear failure tests of coal-like rock specimens were conducted under the dynamic and constant normal load using the self-developed dynamic static load coupling test system. To analyze the effects of different normal-load levels and disturbances on the strain evolution and failure mechanism of specimens, the load, deformation and AE signals of specimens were monitored during the whole test process.

## 2. Materials and Methods

### 2.1. Specimen Preparation

**Figure 1:**
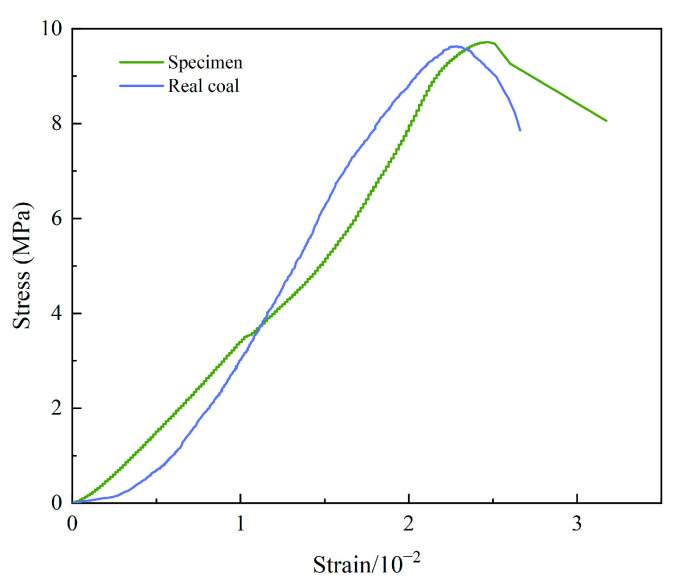
Uniaxial compressive stress–strain curve of real coal and specimen.

Real coal can be damaged in the process of sampling and preparation, which affects the test results. Therefore, coal-like rock materials were made for testing [44]. Their mechanical properties were similar to coal, as shown in Figure 1. The data of real coal were obtained with electro-hydraulic servo testing machine SHT4605. Specimens prepared according to the proportions of Cement: Sand: Plaster = 1:1:0.5. The corresponding quality materials were mixed and poured into molds (100 mm × 100 mm × 100 mm). The blended-material slurry was vibrated and compacted. After 24 h, all specimens were removed from the molds. The specimens were placed in curing boxes to cure for 28 d. Then, the specimens with significant discreteness of sound velocity were removed by ultrasonic testing. The tests were conducted after 28 days of curing.

### 2.2. Testing Equipment

**Figure 2:**
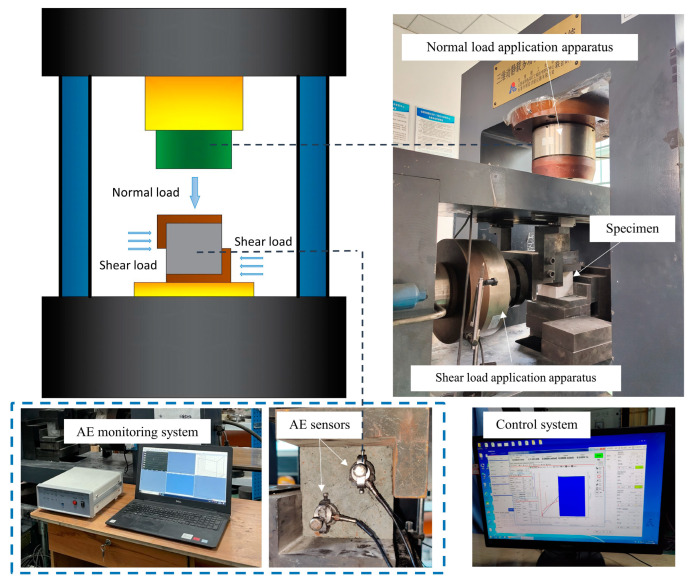
Testing system.

The independent developmental dynamic and static load coupling electro-hydraulic servo testing machine was used for the test. Figure 2 shows the testing system. The maximum axial load of the testing machine is 2000 kN. Constant load and dynamic load with different waveforms can be applied independently or simultaneously. The application frequency of dynamic load is 0~20 Hz and the amplitude is 0~60 kN. The maximum shear force is 600 kN.

A DS5-8B acoustic emission detection system was used to monitor the shear failure process of the specimen. The acoustic emission sensor model is RS-2A, the shell material is stainless steel, the detection surface material is ceramic, the frequency range is 50–400 kHz, the center frequency is 150 kHz, and the sampling frequency is 2.5 MHz. The AE sensors were fixed at a diagonal position on the specimen surface. To ensure adequate contact, Vaseline was applied on the surface of the sensor and the specimen. Before the test, the laboratory noise was detected, and the 40 dB threshold value was set to reduce the interference.

### 2.3. Test Procedure

First, the shear tests under constant normal loads of 10 kN, 20 kN, 30 kN, 40 kN, 50 kN and 60 kN were conducted. During the test, the normal load was applied to reach the target value, and then a constant horizontal shear velocity of 100 N/s was applied until the specimen was damaged. Then, in order to study the shear failure mechanical characteristics of the specimen under disturbance, the frequency of the dynamic normal load was 5 Hz [45], and the shear tests under 10 kN, 30 kN and 50 kN dynamic normal loads were performed. In the test, the normal load was first applied to the target value, and then the sine wave dynamic load (5 Hz, ±10 kN) was applied, and the shear velocity was set to a constant value of 100 N/s. The mechanical characteristics of shear failure of the specimen under different dynamic normal loads are discussed herein. The dynamic normal load can be expressed as:

(1) $$F_{d}=F_{a}sin\left(10\pi t\right)+F_{c}$$

where $F_{d}$ is dynamic normal load, $F_{a}$ is dynamic normal load amplitude(10 kN), t is time and $F_{c}$ is constant normal load.

## 3. Results

### 3.1. Characteristic Analysis of Stress–Strain Curves

**Figure 3:**
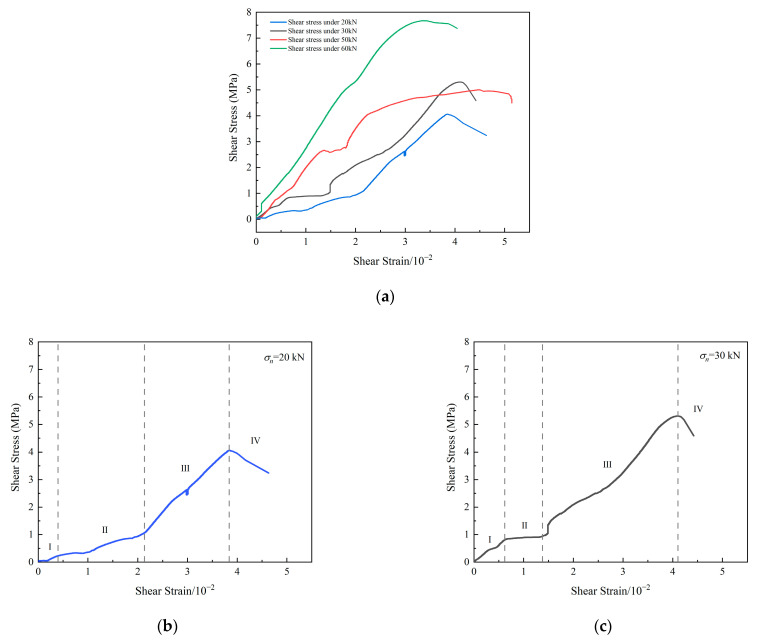

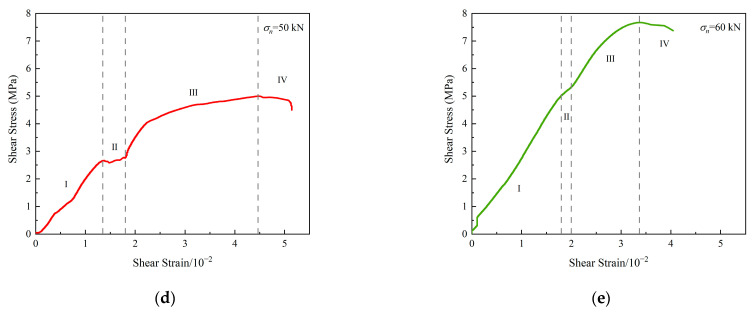
(**a**) Shear stress–strain curves of specimen under constant normal load. (**b**) $\sigma_{n}=20\;\mathrm{kN}$. (**c**) $\sigma_{n}=30\;\mathrm{kN}$. (**d**) $\sigma_{n}=50\;\mathrm{kN}$. (**e**) $\sigma_{n}=60\;\mathrm{kN}$.

As shown in Figure 3, according to the slope of the curve, the shear stress–strain curve was divided into four stages: crack initial compaction stage (I), shear slip stage (II), elastic–plastic stage (III) and post-peak stage (IV).

Early in the first stage, the slope of the curve was small, and the curve was concave. At this moment, the original micro fissures in the specimen were gradually closed under the action of external force. At the later of this stage, the curve changed approximately linearly. As the shear load continued to increase, the initial cracks inside the specimen began to develop.

With the increase in shear load, the shear stress–strain curve entered the shear slip stage. The slope of the curve became smaller. There were platforms on the curve when the normal load was 20 kN, 30 kN and 50 kN. At this stage, the ability of the specimen to resist tangential deformation became weak. The reason was that the normal deformation of the specimen increased under the action of shear load due to the dilation effects [46]. Since the normal force was constant, the normal deformation could not be limited. Therefore, with the growth and coalescence of cracks inside the specimen, shear slip surfaces appeared in specimen. Thus, in the shear slip stage, the tangential deformation of the specimen was large when the shear load increased little. Furthermore, with the increase in normal load, only if the specimen was subjected to greater shear load did the curve enter the shear slip stage and the shear slip stage of the shear stress–strain curve become shorter.

In the elastic–plastic stage, the shear stress–strain curve was nonlinear. After the specimen shear slipped, with the increase in shear load, the cracks in the specimen were compacted again. During the shearing process, there were oblique cracks in the specimen. Under the action of constant normal load and shear load, oblique cracks were compacted and failure surface slipped. Therefore, the slope of the curve was variable. This stage is not easy to divide, and more detailed research is in progress. Finally, when the shear stress reached the peak, the internal cracks of the specimen were penetrated. At this moment, the slope of the curve was 0.

In the post-peak stage, under the action of normal load, there was friction on the failure surface inside the specimen. Therefore, the specimen can still resist the shear force.

**Figure 4:**
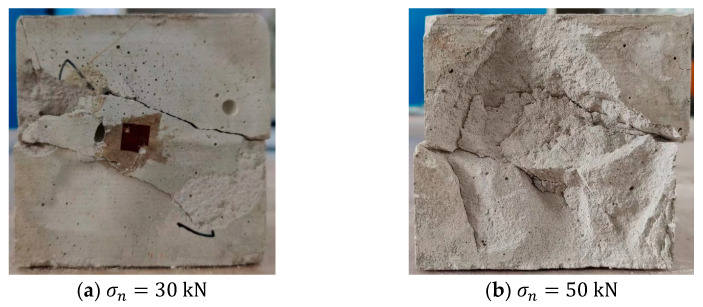
(**a**) Failure shape of specimen under 30 kN normal load. (**b**) Failure mode of specimen under 50 kN normal load.

Figure 4 shows the shear failure shapes of specimens under 30 kN normal load and 50 kN normal load. When the normal load was 30 kN, there were only two main cracks on the surface of the specimen. When the normal load was 50 kN, more oblique cracks appeared on the specimen surface, which may be the reason that the shear strength of $\sigma_{n}=50\;\mathrm{kN}$ was lower than the shear strength of $\sigma_{n}=30\;\mathrm{kN}$. Therefore, there is an overlapping of the curves representing the 30 kN and 50 kN loads in Figure 3a.

### 3.2. Effect of Dynamic Normal Load on Shear Strength

**Figure 5:**
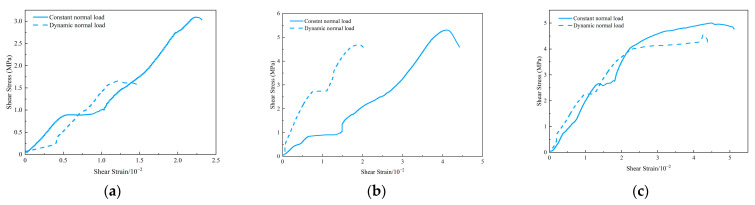
Shear stress–strain curves under different normal loads. Normal loads of (**a**) 10 kN, (**b**) 30 kN and (**c**) 50 kN.

**Figure 6:**
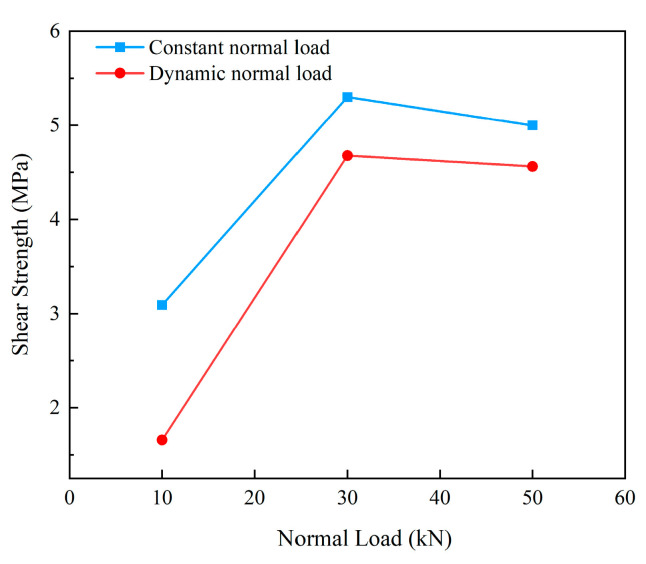
Relationship between normal load and shear strength.

It has been found that cracks and voids will reduce the elastic modulus of rock [47], and the variation in the shear modulus of sandstone is closely related to the types and distribution of cracks [48]. As the shear load increased, the micro fissures in the specimens were compacted and gradually closed. The collective effects of dynamic normal load and shear load further promoted the changes in the fractures in the specimen, thus leading to the changes in the mechanical properties of the specimen. Figure 5 shows the shear stress–strain curve of the shear failure process under the coupling action of constant normal load (10 kN, 30 kN, 50 kN) and dynamic normal load (±10 kN). It can be seen that the strength and deformation characteristics of the specimens were closely related to the normal load. Peak shear displacement increased with normal load. The coupling action of dynamic normal load and constant normal load at different levels decreased the shear strength and peak shear displacement of the specimens compared with the constant normal load. The corresponding shear strengths under the constant normal loads of 10 kN, 30 kN and 50 kN were 3.09 MPa, 5.3 MPa and 4.99 MPa, respectively. While under the coupling action of dynamic normal load and constant normal load, the shear strength under different normal loads decreased to 1.66 MPa, 4.68 MPa and 4.56 MPa, respectively, indicating that the disturbance noticeably weakened the shear strength of the specimen, and reduced the ability of the specimen to resist shear failure. Under the same normal load level, the differences in shear strength under constant load and dynamic load were 1.43 MPa, 0.62 MPa and 0.43 MPa, respectively, as shown in Figure 6, indicating that the influence of disturbance on shear strength weakened with the increase in normal load level. In Figure 5, under the dynamic normal load, the slope of the shear stress–strain curve of the specimen in the elastic deformation stage increased, indicating that the disturbance increased the shear modulus of the specimen. This means that the ability of the specimen to resist shear deformation was enhanced. The reason for the increase in shear modulus is that the coupling action of the dynamic normal load and the shear load changed the state of the original fractures and new fractures in the specimen, further leading to the increase in the shear modulus of the specimen.

### 3.3. Normal Displacement Characteristics

**Figure 7:**
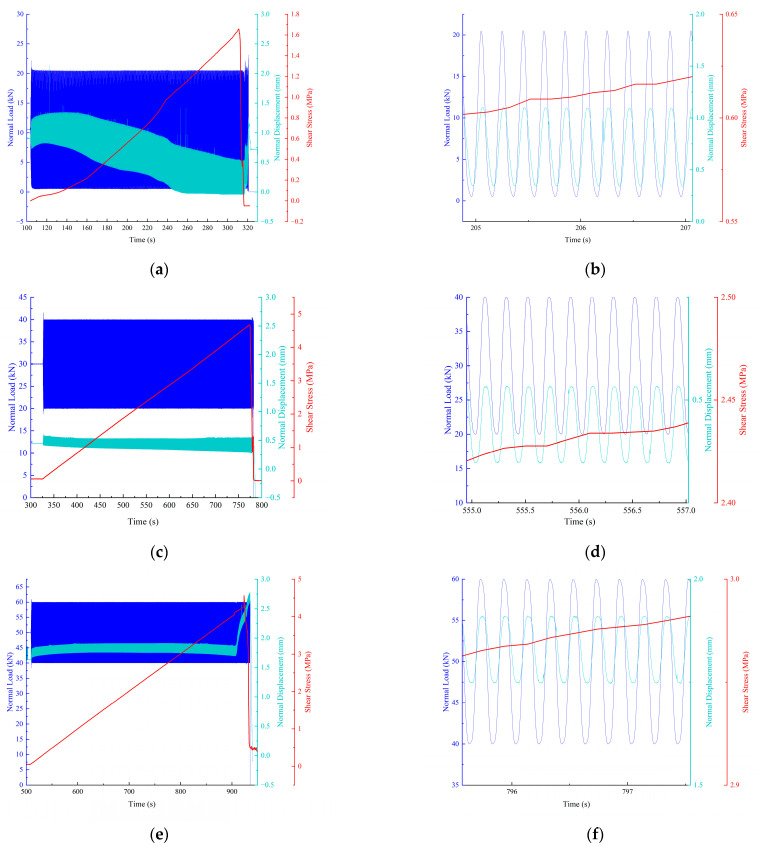
Shear stress, normal displacement and normal load with time under different dynamic normal loads. (**a**) Under 10 kN dynamic normal load. (**b**) Partial enlarged view of (**a**). (**c**) Under 30 kN dynamic normal load. (**d**) Partial enlarged view of (**c**). (**e**) Under 50 kN dynamic normal load. (**f**) Partial enlarged view of (**e**).

As shown in Figure 7, with the variation in dynamic normal load, the normal displacement curve also showed a change trend similar to the sinusoidal curve, but there was a time difference between the two waveform changes. The peak value of the displacement change curves at the three normal load levels was about 0.01 s behind the peak value of load. This phenomenon may have been caused by the deformation of particles inside the specimen after being stressed and by the force transfer between particles. By comparing the normal displacement amplitudes of 10 kN, 30 kN and 50 kN, it can be seen that with the increase in the normal load level, the normal displacement amplitude decreased. In this process, the micro fissures and fractures in the specimen were constantly squeezed. Then, before the failure, the normal displacement became smaller and smaller, and the force required for the normal unit displacement of the specimen increased. This means that with the increase in the normal load level, the ability of the specimen to resist the normal deformation increased, and the elastic modulus increased.

### 3.4. Analysis of AE

#### 3.4.1. AE Energy and Cumulative Ring-Down Count

Scholars have studied the material damage and fracture process by using acoustic emission technology, and believe that ring-down count can reflect the change of material properties well [39], because it is proportional to the strain energy released by the movement of dislocations in the material, the stripping and fracture of inclusions and second-phase particles, and crack growth [49]. The growth of microcracks inside the rock produced AE, which is a ubiquitous phenomenon associated with brittle fracture, and provided a wealth of information regarding the failure process in rock [50]. Ring-down count is a commonly used acoustic emission parameter. When an event hits the sensor, the sensor receives signals. Each oscillation wave of the signal exceeding the threshold is recorded as a ring-down count. The ring-down count is the number of oscillations over the threshold. For the same signal, when the threshold value is different, the ring-down count will be different [51]. In this test, the threshold was set to 40 dB. This research mainly analyzed the AE ring-down count and AE energy. To a certain extent, both can reflect the internal deformation and damage of the specimen. The AE ring-down count reflects the internal damage degree of the specimen, and the AE energy reflects the released energy during the generation and development of the internal fractures of the specimen [31,43].

**Figure 8:**
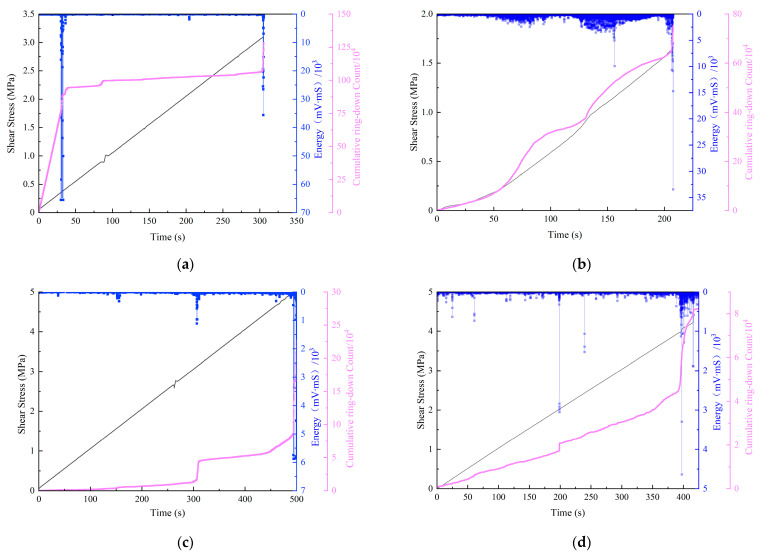
Shear load, AE cumulative ring-down count and energy with time under different normal loads. (**a**) Under 10 kN constant normal load. (**b**) Under 10 kN dynamic normal load. (**c**) Under 50 kN constant normal load. (**d**) Under 50 kN dynamic normal load.

Since the AE curves under 10 kN and 50 kN normal loads are presented well, only the AE data under these two normal loads are described here. As shown in Figure 8, by comparing (a) and (c), during the shear process, the AE cumulative ring-down count under 50 kN constant normal load was significantly lower than that under 10 kN constant normal load. The AE energy under 10 kN constant normal load was more active than that under 50 kN constant normal load, indicating that with the increase in constant normal load level, the new fractures were reduced during the shear process, the expansion of fractures was inhibited, the energy released by damage was less, and the internal damage degree of the specimen was also lessened. By comparing (a) with (b) and (c) with (d), it can be seen that during the shear process, the AE energy under the dynamic normal load was more active, indicating that in the shear failure process, the dynamic normal-load-induced damage inside the specimen was more serious than that under the constant normal load, resulting in AE events that were more active and had greater releases of energy. When the specimen was close to shear failure, the amplification of AE cumulative ring-down count under 10 kN and 50 kN dynamic normal loads were 98,610 and 34,573, respectively, while the amplification of AE cumulative ring-down count under 10 kN and 50 kN constant normal loads were 216,872 and 140,092, respectively. AE cumulative ring-down count under dynamic normal load was significantly less than that under constant normal load, indicating that the severity of fracture in the specimen at the last moment under dynamic load was weakened. This was consistent with the foregoing evidence that most of the damage occurs in the shear process, when the specimen is under the dynamic normal load. The energy is mainly released during the shear failure process, and the AE response at the last moment is weak.

**Figure 9:**
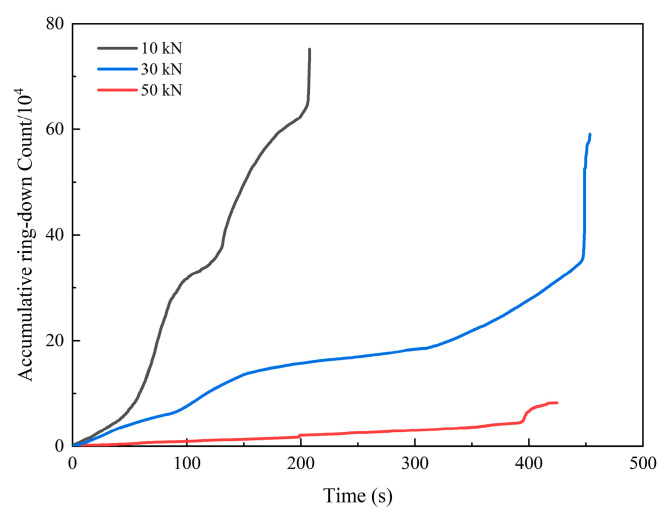
The curve of AE cumulative ring-down count with time under different dynamic normal loads.

Figure 9 shows the curve of the AE cumulative ring-down count with time under the action of 10 kN, 30 kN and 50 kN dynamic normal loads. It can be seen that the curves were rising, but there was an alternation of gentle and rapid rise. Under three different dynamic normal load levels, the relationship between the AE cumulative ring-down count of the specimens was 10 kN > 30 kN > 50 kN, but the loading time was irregular. Among them, the AE cumulative ring-down count curve under 10 kN dynamic normal load was the steepest and the gentle stage was the shortest, the AE cumulative ring-down count curve under 50 kN dynamic normal load was the gentlest and the steep stage was not obvious and the AE cumulative ring-down count curve under 30 kN dynamic normal load was between them. This means that the smaller the dynamic normal load is, the faster the fractures propagate and the more fiercely the energy releases during the shear process.

#### 3.4.2. Analysis of Damage Evolution

Many studies have proved that the AE energy is closely related to the damage of coal-rock mass, and it is directly proportional to the propagation and coalescence of fractures and the release of energy. Therefore, this study used AE energy to describe the damage characteristics of coal-like rock specimens.

L. M. Kachanov defined the damage variable as:

(2) $$D=\frac{A_{d}}{A}$$

where D is the damage variable, $A_{d}$ is the damaged area of the cross section of the specimen and A is cross sectional area of specimen without damage.

Cumulative AE energy released by the specimen from initial condition to failure is E. AE energy released by unit area is $E_{w}$.

(3) $$E_{w}=\frac{E}{A}$$

When the damaged area is $A_{d}$, cumulative AE energy is $E_{d}$.

(4) $$E_{d}=E_{w}A_{d}=\frac{E}{A}A_{d}$$

Therefore, the relationship between cumulative AE energy and damage variables is as follows:

(5) $$D=\frac{E_{d}}{E}$$

**Figure 10:**
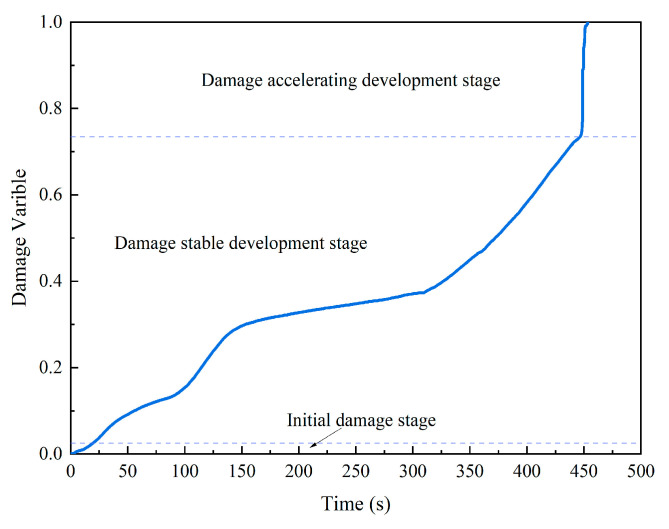
The curve of damage-variable time under a 30 kN dynamic normal load.

In fact, the specimen was damaged before the shear load was applied due to the normal load. In order to analyze the damage evolution process of shear under dynamic normal load, it is assumed that the specimen was not damaged before the shear load was applied. The damage variable was calculated by Equation (5). The damage-variable time curve is shown in the Figure 10. Because the shapes of the damage-variable time curve under different normal loads were similar, only the damage-variable time curve under 30 kN dynamic normal load is shown here.

As shown in the Figure 10, the damage evolution process of the specimen can be divided into three stages in the shear failure process under dynamic normal load. The first was the initial damage stage. This stage was short, the curve growing slowly because the fractures in the specimen were compressed and closed, and no new fractures were generated. The second was the damage stable development stage. This stage took the longest time, and damage evolution was stable. The last was the damage accelerating development stage. This stage was shortest, but the damage evolution was the fastest.

#### 3.4.3. Analyzing the Failure Mechanism by RA-AF

The average frequency (AF) is defined as the ring-down count/duration in the AE signal parameters, and the RA value is the rise time/amplitude. The radio of RA and AF can be used to judge the fracture mode in the rock failure process [52]. Gan [53] used the moment tensor inversion module in Insite to calculate the shear fracture ratio in the rock failure process, estimated the k value (AF/RA) in the test and obtained the recommended value. When k=90, the proportion of shear fracture was the most similar to the result judged by RA-AF. When k<90, the fracture was shear fracture. When k>90, the fracture was tension or mixed fracture.

**Figure 11:**
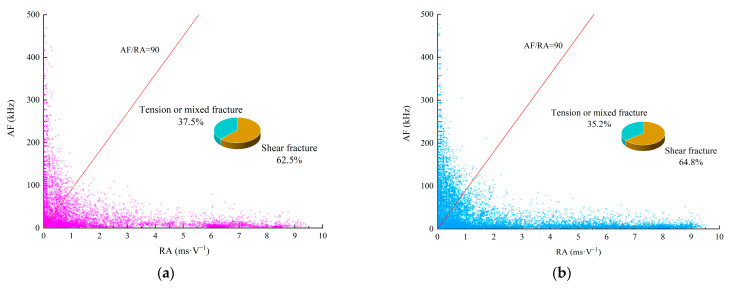

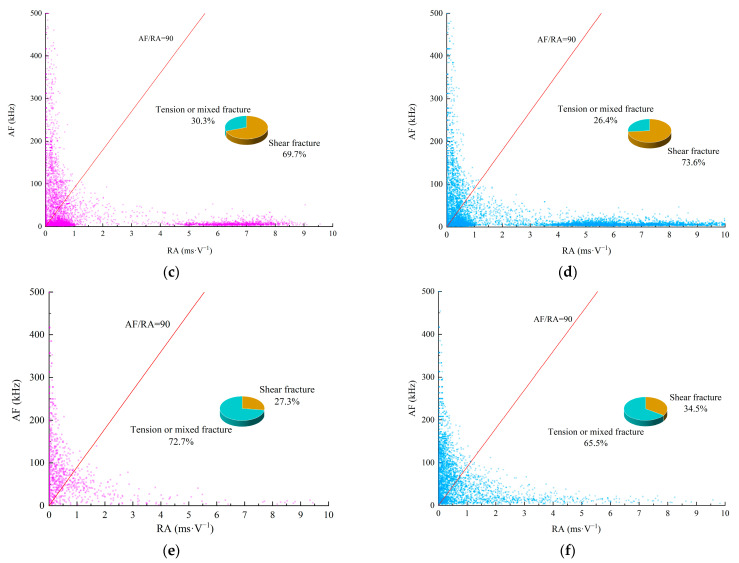
The change process of fracture mode at different stages under dynamic normal load. (**a**) Elastic stage under 10 kN. (**b**) Unstable development fracture stage under 10 kN. (**c**) Elastic stage under 30 kN. (**d**) Unstable development fracture stage under 30 kN. (**e**) Elastic stage under 50 kN. (**f**) Unstable development fracture stage under 50 kN.

In Figure 11, k=90 is a split line, the AE data points below the split line are shear fracture signals, and the data points above the split line are tension or mixed fracture signals. It can be seen that under the coupling action of dynamic normal loads and constant normal loads, the fractures produced in different stages of the shear failure process included both shear fractures and tension or mixed fractures, and the differences between the two stages were obvious. In the elastic deformation stage, when the dynamic normal load was 10 kN, there were more AE signals (k<90), accounting for 62.5%, indicating that shear fractures were the main fracture. When the dynamic normal load was 30 kN, the proportion of shear fracture in the elastic stage increased to 69.7%. When the dynamic normal load was 50 kN, there were more AE signals (k>90), accounting for 72.7%, indicating that the great majority of fractures fell within the tensile or mixed fracture category.

In the unstable development fracture stage, there was a similar law with the elastic deformation stage. When the dynamic normal load was 10 kN, the AE signal (k<90) accounted for 64.8%, indicating that shear fracture was the main fracture. When the dynamic normal load was 30 kN, the proportion of shear fracture in the fracture increased to 73.6%. It was found that when the dynamic normal load was 10 kN or 30 kN, the great majority of fractures fell within the shear fracture category. However, when the dynamic normal load was 50 kN, the AE signal (k>90) accounted for 65.5%, indicating that largely of fractures fell within the tensile or mixed fracture category.

In the unstable development fracture stage, the proportion of shear failure increased by 2.3%, 3.9% and 7.2%, respectively, compared with the elastic deformation stage. In the unstable development fracture stage, when the dynamic normal load was 10 kN or 30 kN, fractures largely fell within the shear fracture category. However, when the dynamic normal load was 50 kN, although the proportion of shear fractures increased, the most numerous fractures were still tensile or mixed fractures.

## 4. Conclusions

In this study, a series of shear tests under dynamic normal load and constant normal load were conducted to investigate the influence of disturbance on the shear mechanical properties of coal-like rock materials. The failure process and damage evolution were studied by analyzing AE parameters. The conclusions are summarized as follows:

1. Under the action of constant normal load, the shear failure process of a specimen can be divided into four stages: crack initial compaction stage (I), shear slip stage (II), elastic-plastic stage (III) and post-peak stage (IV). At the shear slip stage, there are shear slip surfaces inside the specimen.
2. Disturbance has a great influence on the mechanical properties and failure mechanism of coal-like rock materials in the shear failure process. Disturbance reduces the shear strength of sandstone and improves the shear modulus of coal-like rock materials. The influence of disturbance on the shear strength of a specimen decreases with the increase in normal load.
3. By analyzing AE data, it can be found that the dynamic normal load makes the internal damage of a specimen more serious in the shear failure process, and the severity of final failure is reduced. With the increase in dynamic normal load, the fracture propagation speed becomes slower and less energy is released during the shear process.
4. Based on the AE cumulative energy, the damage variable of shear process under dynamic normal load was derived, the damage evolution process was divided into three stages, and the change characteristics of different stages were analyzed.
5. The shear failure mechanism of specimens was judged by RA-AF. When the dynamic normal load was 10 kN or 30 kN, the fractures in the shear failure process were mainly shear fractures. When the dynamic normal load was 50 kN, the fractures were mainly tensile or mixed fractures.

General remark: Due to the limitation of the quantity of specimens, shear tests under only three different dynamic normal loads were conducted. Therefore, the conclusions may have limitations. More shear tests under dynamic normal load must be conducted to study the influence of normal disturbance on the mechanical properties of coal-rock mass.

## Data Availability

The data used to support the findings of this study are available from the corresponding author upon reasonable request.

## References

[1] Vižintin G., Kocjančič M., Vulić M. (2016). Study of Coal Burst Source Locations in the Velenje Colliery. Energies.

[2] Holub K. (2007). A study of mining-induced seismicity in czech mines with longwall coal exploitation. J. Min. Sci..

[3] Li T., Cai M.F., Cai M. (2007). A review of mining-induced seismicity in China. Int. J. Rock Mech. Min. Sci..

[4] Cai M., Kaiser P. (2006). Visualization of rock mass classification systems. Geotech. Geol. Eng..

[5] Tibbett J., Suorineni F., Hebblewhite B. (2016). Preliminary investigation of rockmass response to undercut blasting in a block cave mining system using VRSV. CIM J..

[6] He M.C., Ren F.Q., Liu D.Q. (2018). Rockburst mechanism research and its control. Int. J. Min. Sci. Technol..

[7] Li X.B., Gong F.Q. (2021). Research progress and prospect of deep mining rock mechanics based on coupled static-dynamic loading testing. J. China Coal Soc..

[8] Li X.B., Zhou J., Wang S.F., Liu B. (2017). Review and practice of deep mining for solid mineral resources. Chin. J. Nonferrous Met..

[9] Xie H.P. (2019). Research review of the state key research development program of China: Deep rock mechanics and mining theory. J. China Coal Soc..

[10] Liang C.Y., Li X., Li S.D., Hao J.M., Ma C.F. (2012). Study of strain rates threshold value between static loading and quasi-dynamic loading of rock. Chin. J. Rock Mech. Eng..

[11] Tang L.Z., WU J.L., Liu T., Zhu J., Shu J.B. (2014). Mechanical experiments of marble under high stress and cyclic dynamic disturbance of small amplitude. J. Cent. South Univ. (Sci. Technol.).

[12] Zhang Q.B., Zhao J. (2014). A Review of Dynamic Experimental Techniques and Mechanical Behaviour of Rock Materials. Rock Mech. Rock Eng..

[13] Gao M.Z., Wang M.Y., Xie J., Gao Y.N., Deng G.D., Yang B.G., Wang F., Hao H.C., Xie H.P. (2020). In-situ disturbed mechanical behavior of deep coal rock. J. China Coal Soc..

[14] Gong F.Q., Li X.B., Liu X.L. (2010). Experimental study of dynamic characteristics of sandstone under one-dimensional coupled static and dynamic loads. Chin. J. Rock Mech. Eng..

[15] Gong F.Q., Li X.B., Liu X.L. (2011). Preliminary experimental study of characteristics of rock subjected to 3D coupled static and dynamic loads. Chin. J. Rock Mech. Eng..

[16] Gong F.Q., Lu D.H., Li X.B., Rao Q.H., Fu Z.T. (2014). Toughness increasing or decreasing effect of hard rock fracture with pre-static loading under dynamic disturbance. Chin. J. Rock Mech. Eng..

[17] Li X.B., Zuo Y.J., Ma C.D. (2006). Constitutive model of rock under coupled static-dynamic loading with intermediate strain rate. Chin. J. Rock Mech. Eng..

[18] Liu E.L., He S.M. (2012). Effects of cyclic dynamic loading on the mechanical properties of intact rock samples under confining pressure conditions. Eng. Geol..

[19] Bagde M.N., Petros V. (2005). Fatigue properties of intact sandstone samples subjected to dynamic uniaxial cyclical loading. Int. J. Rock Mech. Min. Sci..

[20] Carter J.P., Ooi L.H. (1987). A Constant Normal Stiffness Direct Shear Device for Static and Cyclic Loading. Geotech. Test. J..

[21] Zhang L., Thornton C. (2007). A numerical examination of the direct shear test. Géotechnique.

[22] Xu J., Liu J., Wu H., Cheng L.C., Lu L.F. (2013). Test study of sandstone cracking and propagation process under compressive-shear stress. Chin. J. Rock Mech. Eng..

[23] Xu J., Lu L.F., Yang H.W., Zhang Y., Wang L. (2011). Study of evolution law of microfracturing progress of sandstone under shear loading. Chin. J. Rock Mech. Eng..

[24] Cheng T., Guo B.H., Sun J.H., Tian S.X., Sun C.X., Chen Y. (2022). Establishment of constitutive relation of shear deformation for irregular joints in sandstone. Rock Soil Mech..

[25] Huang D., Cen D.F., Song Y.X. (2020). Comparative Investigation on the Compression–Shear and Tension–Shear Behaviour of Sandstone at Different Shearing Rates. Rock Mech. Rock Eng..

[26] Dang W.G., Konietzky H., Frühwirt T. (2016). Direct shear behavior of a plane joint under dynamic normal load (DNL) conditions. Eng. Geol..

[27] Dang W.G., Konietzky H., Frühwirt T. (2017). Direct Shear Behavior of Planar Joints Under Cyclic Normal Load Conditions: Effect of Different Cyclic Normal Force Amplitudes. Rock Mech. Rock Eng..

[28] Li S.G., Bie C.F., Zhao P.X., Li L., Lin H.F. (2017). Study on influence factors of new solid-gas coupling simulation material. J. Min. Saf. Eng..

[29] Li Q.G., Lin B.Q., Zhai C. (2014). The effect of pulse frequency on the fracture extension during hydraulic fracturing. J. Nat. Gas Sci. Eng..

[30] Zhai C., Xu J.Z., Liu S.M., Qin L. (2018). Fracturing mechanism of coal-like rock specimens under the effect of non-explosive expansion. Int. J. Rock Mech. Min. Sci..

[31] Liu B.X., Huang J.L., Wang Z.Y., Liu L. (2009). Study on damage evolution and acoustic emission character of coal-rock under uniaxial compression. Chin. J. Rock Mech. Eng..

[32] Carlson S.R., Young R.P. (1993). Acoustic emission and ultrasonic velocity study of excavation-induced microcrack damage at the underground research laboratory. Int. J. Rock Mech. Min. Sci. Geomech. Abstr..

[33] Liu D.W., Jiang S.L., Tang Y. (2022). Mechanical Properties of Metasandstone under Uniaxial Graded Cyclic Loading and Unloading. Appl. Sci..

[34] Zhao C.X., Liu J.F., Lyu C., Chen W.Z., Li X.Y., Li Z.C. (2022). Experimental study on mechanical properties, permeability and energy characteristics of limestone from through-coal seam (TCS) tunnel. Eng. Geol..

[35] Cheon D.S., Jung Y.B., Park E.S., Song W.K., Jang H.L. (2011). Evaluation of damage level for rock slopes using acoustic emission technique with waveguides. Eng. Geol..

[36] Meng F.Z., Wong L.N.Y., Zhou H., Wang Z.Q., Zhang L.M. (2020). Asperity degradation characteristics of soft rock-like fractures under shearing based on acoustic emission monitoring. Eng. Geol..

[37] Aggelis D.G., Mpalaskas A.C., Matikas T.E. (2013). Acoustic signature of different fracture modes in marble and cementitious materials under flexural load. Mech. Res. Commun..

[38] Zha E.S., Zhang R., Zhang Z.T., Ai T., Ren L., Zhang Z.P., Liu Y., Lou C.D. (2020). Acoustic Emission Characteristics and Damage Evolution of Rock under Different Loading Modes. Energies.

[39] Wu S., Qin G.P., Cao J. (2022). Deformation, Failure, and Acoustic Emission Characteristics under Different Lithological Confining Pressures. Materials.

[40] Aggelis D.G. (2011). Classification of cracking mode in concrete by acoustic emission parameters. Mech. Res. Commun..

[41] Wang H.J., Liu D.A., Cui Z.D., Cheng C., Jian Z. (2016). Investigation of the fracture modes of red sandstone using XFEM and acoustic emissions. Theor. Appl. Fract. Mech..

[42] Ohno H., Ohtsu M. (2010). Crack classification in concrete based on acoustic emission. Constr. Build. Mater..

[43] Zhou Z.L., Li G.N., Ning S.L., Du K. (2014). Acoustic emission characteristics and failure mechanism of high-stressed rocks under lateral disturbance. Chin. J. Rock Mech. Eng..

[44] Zhai C., Zheng Y.F., Yu X., Xu J.Z., Sun Y., Cong Y.Z., Tang W., Li Y.J., Zhu X.Y., Chen A.K. (2020). Experimental study on the mechanical properties of coal-like materials for hydraulic fracturing simulation. Coal Geol. Explor..

[45] Wen X.Z., Feng G.R., Guo J., Wang P.F., Qian R.P., Zhu L.J., Hao C.L., Fan Y.J. (2022). Dynamic tensile mechanical response properties of sandstone under medium and low strain rate disturbance load. Chin. J. Rock Mech. Eng..

[46] Yin Q., Jing H.W., Meng B., Liu R.C., Wu J.Y., Wu Y.J. (2021). Macroscopic and microscopic shear mechanical properties of sandstone under CNL and CNS boundary conditions. J. Min. Saf. Eng..

[47] Mavko G., Jizba D. (2012). Estimating grain-scale fluid effects on velocity dispersion in rocks. Geophysics.

[48] Li D.Q., Si W.P., Chen S.H., Wei J.X., Di B.R. (2021). Experimental study and theoretical simulation of dynamic shear modulus hardening in saturated tight sandstone. Chin. J. Geophys..

[49] Liu X.W., Lin J.Z., Yuan Z.Y. (1997). Research on evaluation of material fatigue damage by acoustic emission technology. China Railw. Sci..

[50] Lockner D. (1993). The role of acoustic emission in the study of rock fracture. Int. J. Rock Mech. Min. Sci. Geomech. Abstr..

[51] Brindley B.J., Holt J., Palmer I.G. (1973). Acoustic emission-3: The use of ring-down counting. Non-Destr. Test..

[52] Ramirez-Jimenez C.R., Papadakis N., Reynolds N., Gan T.H., Purnell P., Pharaoh M. (2004). Identification of failure modes in glass/polypropylene composites by means of the primary frequency content of the acoustic emission event. Compos. Sci. Technol..

[53] GAN Y.X., Wu S.C., Ren Y., Zhang G. (2020). Evaluation indexes of granite splitting failure based on RA and AF of AE parameters. Rock Soil Mech..

